# Sex differences in heart rate responses to postural provocations

**DOI:** 10.1016/j.ijcard.2019.09.044

**Published:** 2019-12-15

**Authors:** Katerina Hnatkova, Martina Šišáková, Peter Smetana, Ondřej Toman, Katharina M. Huster, Tomáš Novotný, Georg Schmidt, Marek Malik

**Affiliations:** a National Heart and Lung Institute, Imperial College, 72 Du Cane Road, Shepherd’s Bush, London, W12 0NN, England, UK; b Department of Internal Medicine and Cardiology, University Hospital Brno, Faculty of Medicine, Masaryk University, Jihlavská 20, 625 00, Brno, Czech Republic; c Wilhelminenspital der Stadt Wien, Montleartstraße 37, 1160, Vienna, Austria; d Klinikum rechts der Isar, Technische Universität München, Ismaninger Straße 22, D-81675, Munich, Germany

**Keywords:** Sex differences, Postural changes, Heart rate, Heart rate variability, Autonomic modulations, Autonomic responsiveness, Responses to stress

## Abstract

Sex differences are known in several facets of cardiac electrophysiology, mostly concerning myocardial repolarisation. In this study, heart rate and heart rate variability (HRV) responses to postural provocations were compared in 175 and 176 healthy females and males, respectively (aged 33.1 ± 9.1 years). Two different postural provocative tests with position changes supine→sitting→standing→supine and supine→standing→sitting→supine (15-min standing, 10-min other positions) were performed up to 4 times in each subject. Heart rate and heart rate variability spectral indices were measured in 5-min windows before positional changes. At supine position, females had averaged heart rate approximately 5 beats per minute (bpm) faster than males and this sex difference was practically constant during the postural changes. In both sexes, change supine→sitting and supine→standing increased heart rate by approximately 10 and 30 bpm, respectively, with no statistical differences between the sex groups. At supine baseline, females had normalised high frequency components (nHF) of HRV approximately 7% larger compared to males (p < 0.001). While the same difference in nHF was found at sitting, the change to standing position lead to significantly larger nHF reduction in females compared to males (mean changes 22.5 vs 17.2%, p < 0.001). This shows that despite similar heart rate increase, females respond to standing by more substantial shifts in cardiac sympatho-vagal modulations. This makes it plausible to speculate that the differences in autonomic reactions to stress contribute to the known sex-differences in psychosocial responses to stressful situations and to the known difference in susceptibility to ventricular fibrillation between females and males.

## Highlights

• Postural change supine to sitting increases heart rate by 10 beats per minute in females and males.
• Postural change supine to standing increases heart rate by 30 beats per minute in females and males.
• Females respond to the supine to standing change by more pronounced decrease in nHF of HRV.
• This might partially explain the differences in female and male responses to stress.

## 1 Introduction

Incidence of different cardiovascular pathologies and their clinical manifestations are well known to differ between women and men. In cardiac electrophysiology, this includes less frequent ventricular fibrillation [1] and more frequent torsade de pointes [2] in pre-menopausal women. Important studies investigated sex differences in electrophysiologic background, mainly concentrating on ventricular repolarisation [[3], [4], [5]].

Somewhat lesser attention has been paid to the sex differences in cardiac autonomic modulations although it is also known that autonomic control plays a pivotal role in arrhythmogenesis [6,7]. Several non-invasive studies of heart rate variability (HRV) described increased high frequency and lowered low frequency modulations in resting women compared to men [[8], [9], [10]] but other studies failed to find such differences between sexes [11,12]. Little is known about sex differences in autonomic responses to provocations although such reactions might also play a role in arrhythmogenesis [13].

To contribute to this aspect of sex differences in autonomic modulations, we have used Holter recordings of a pharmacological study conducted in healthy subjects of both sexes. Per study protocol, the subjects repeatedly changed their positions which allowed us to study their heart rate and HRV responses to postural provocations.

## 2 Methods

### 2.1 Investigated population

The available data were originated from a large clinical pharmacology study conducted in healthy subjects. Repeated 12-lead day-time Holter recordings were made in all study subjects while they were on no treatment. During the complete clinical pharmacology study, the subjects were free of alcohol and/or caffeinated drinks ingestion. All subjects had a normal screening electrocardiogram and normal clinical investigation usual in clinical pharmacology studies [14]. The original study was approved by the relevant ethics boards and all participants gave written informed consent in accordance with the Helsinki declaration. Since only anonymised off-treatment data are presented here, the details of the source study are of no relevance.

Female subjects of the study had a negative pregnancy test and for the duration of the clinical pharmacology study were not on hormonal contraceptives. Body heights and weights of the subjects were measured at screening of the source study. Body mass index (BMI) was calculated as *w*/*h*^2^ where *w* is the body weight in kilograms and *h* is the body height in metres.

### 2.2 Investigative protocol

Per study protocol, drug-free recordings were obtained at 4 different days within a 25-day period. At each of these days, the subjects followed 2 different procedures of postural provocations. During the first procedure (test 1), 10-min strict supine position was followed by 10-min unsupported sitting position, followed by 15-min unsupported standing position, followed by 10-min strict supine position. During the second procedure (test 2), the standing and sitting positions were reversed, i.e. a 10-min strict supine position was followed by 15-min standing position, followed by 10-min sitting position, followed by 10-min strict supine position. Postural position changes were made actively by the study subject with the per-protocol instruction to achieve the new body position in no more than 20 s.

During the provocative tests, the study subjects had no contact with each other, were not allowed to speak and while in the prescribed positions, were instructed to make no body movements apart from shallow breathing.

On each day, both tests were performed in the afternoon separated by approximately 1 h gap. During these days, the subjects did not have any breakfast and consumed a light lunch approximately 4 h prior to the first postural test. They were allowed to drink water and/or zero-caloric non-caffeinated drinks between the lunch and the first test and between the tests. The subjects were not allowed to smoke at least 24 h before each of the investigation days.

Data of an entire postural provocative test were excluded from the analysis if the subject was unable to complete the test because of pre-syncopal symptoms, substantial discomfort, or other reasons that prevented the test completion. Subjects who did not complete one of the tests were still included in the population of the other test of the same day and in the postural test of subsequent investigation days unless they dropped out from the source study in the meantime.

### 2.3 Electrocardiographic measurements

Continuous 12-lead Holter recordings with electrodes in Mason-Likar positions were obtained during the study days. Their span fully covered the episodes of the postural provocative tests. Using previously described measurement procedures [15] individual QRS complexes were identified and classified as belonging to sinus rhythm or corresponding to supraventricular or ventricular ectopic beats. Series of RR intervals measured at a 1 kHz resolution were obtained with systematic timing of QRS complexes achieved by calculating their maximum cross-correlations.

### 2.4 Heart rate and heart rate variability

For the purposes of tracking heart rate changes, heart rate was measured in 10-s windows that were moved through the continuous recordings in 5-s steps.

To study the autonomic responses, 5-min intervals were obtained between 4.5 and 9.5 min of each postural position as well as between 9.5 and 14.5 min of the standing positions. This eliminated the transition periods during which the heart rate was unstable because of postural changes. In each of these non-overlapping 5-min intervals, heart rate was obtained from the averaged RR interval durations. Subsequently, in each of these 5-min intervals, the sequence of RR intervals was detrended and Blackman-Tukey modification of Fast Fourier transformation was used to obtain HRV spectra, providing total power, and low frequency (LF, 0.04–0.15 Hz) and high frequency (HF, 0.15–0.4 Hz) power components [16].

Since the absolute measurements of the LF and HF frequency components of HRV cannot be meaningfully compared between episodes that differ substantially in the underlying heart rate, quasi-normalised nLF = LF/(LF + HF) and nHF = HF/(LF + HF) components were obtained. For each analysed interval, nLF + nHF = 1. Therefore, for data evaluation, only nHF measurements were used.

### 2.5 Statistics and data presentation

Continuous data are presented as mean ± standard deviation. Where appropriate, graphic displays show data means with dual-sided 95% confidence intervals derived assuming normal distribution. Corresponding measurements in females and males were compared using standard t-tests assuming different standard deviations. Cumulative density distributions were compared by Kolmogorov-Smirnov tests. Dependencies of electrocardiographic measurements on age and on underlying heart rate were studied by means of linear regressions calculated together with 95% confidence interval bands. The slopes of the linear regressions between females and males were compared. P-values below 0.05 were assumed statistically significant. Because of mutual interdependency of different measurements, no adjustment for multiplicity of testing was made. Statistical analyses were performed using SPSS Statistics version 25 (IBM Corporation, Armonk, NY, USA).

## 3 Results

### 3.1 Population

The original study enrolled 176 females and 176 males. Because of study drop-outs, both tests 1 and 2 were performed 672 and 680 times in females and males, respectively. Valid data of postural test 1 (supine → sitting → standing → supine) were available 4, 3, 2, and 1 times in 100, 42, 21, and 9 females, respectively. In males, the corresponding counts were 112, 40, 21, and 3, respectively. Valid data of postural test 2 (supine → standing → sitting → supine) were available 4, 3, 2, and 1 times in 126, 29, 17, 3 females, respectively. In males, the corresponding counts were 128, 28, 17, and 3, respectively. This means that of all tests 1, valid data were obtained in 557 (85.9%) and 613 (90.1%) cases in females and males, respectively. For test 2, the corresponding numbers were 628 (93.5%) and 633 (93.1%), respectively. No valid postural test data were available in one female (aged 40.1 years).

Hence, the actual population of this study consisted of 175 and 176 healthy females and males, aged 32.6 ± 9.8 and 33.5 ± 8.4 years, respectively (no statistical difference between the female and male ages).

Not surprisingly, females were shorter than males (body height of 1.64 ± 0.07 vs 1.78 ± 0.07 m, p < 0.001) as well as lighter than males (body weight of 67.1 ± 10.0 vs 80.2 ± 10.2 kg, p < 0.001). However, BMI in females (24.8 ± 3.0 kg/m^2^) was not statistically different from that in males (25.4 ± 2.6 kg/m^2^).

### 3.2 Heart rate

**Fig. 1:**
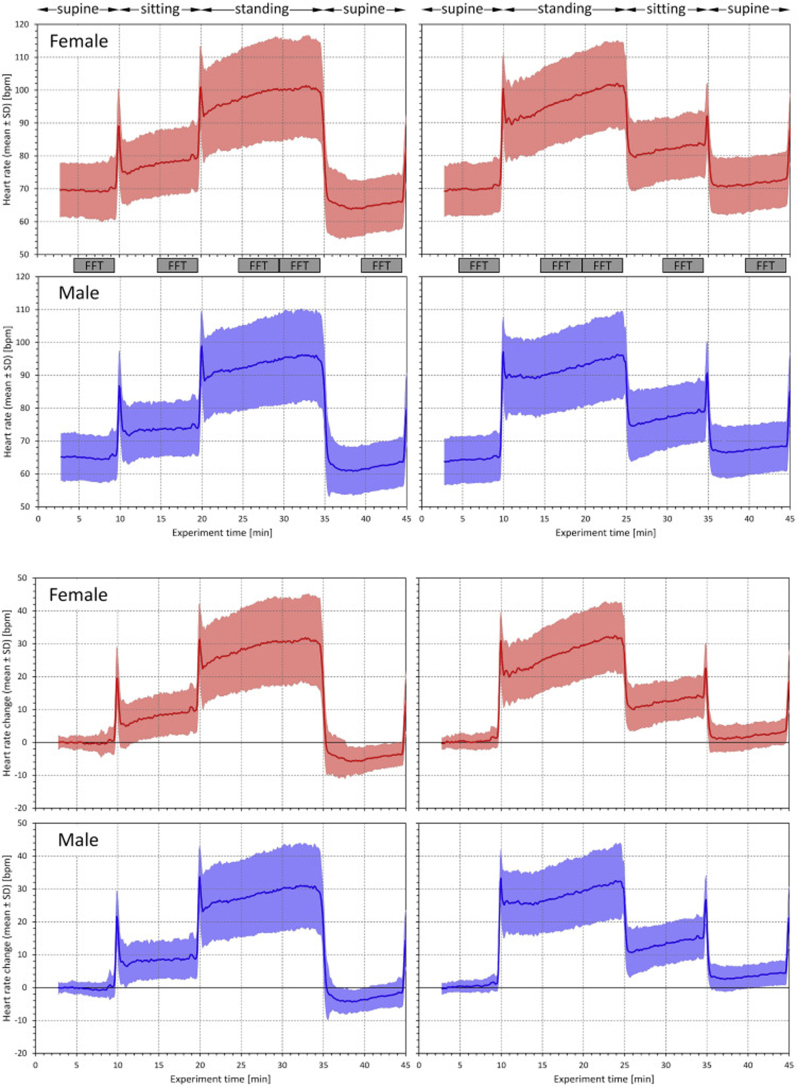
The top part of the Figure shows experiment layout (see the text for details) and heart rate changes during the postural tests. The panels on the left correspond to the postural test 1, the panels on the right to the postural test 2. On top of the panels, postural positions adopted during the test are shown. The grey blocks between the panels indicate the windows during which the 5-min heart rate measurements and the 5-min Fast Fourier transform heart rate variability analyses were made. The top panels show the heart rates measured in females (in red) and in males (in blue). The bottom panels show intra-subject heart rate changes relative to the initial heart rate at the beginning of the starting supine position (red panels for female, blue panels for males). In all panels, the bold lines show the mean heart rate or mean heart rate change while the coloured areas show the bands of ±standard deviation of heart rates or heart rate changes.

Fig. 1 shows the development of heart rates during test 1 and test 2 in females and males. Although, as expected, females had a slightly higher resting heart rate at the beginning of the tests, the intra-individual heart rate response was similar in both sex groups.

During both tests, the heart rate increases from initial supine to the terminal part of the standing period were somewhat unexpectedly large and reached, on average, approximately 30 beats per minute (bpm) in both sex groups.

### 3.3 Heart rate and heart rate variability changes

Heart rate and HRV data in pre-specified 5-min intervals are summarised in Table 1 (in electronic supplement).

**Fig. 2:**
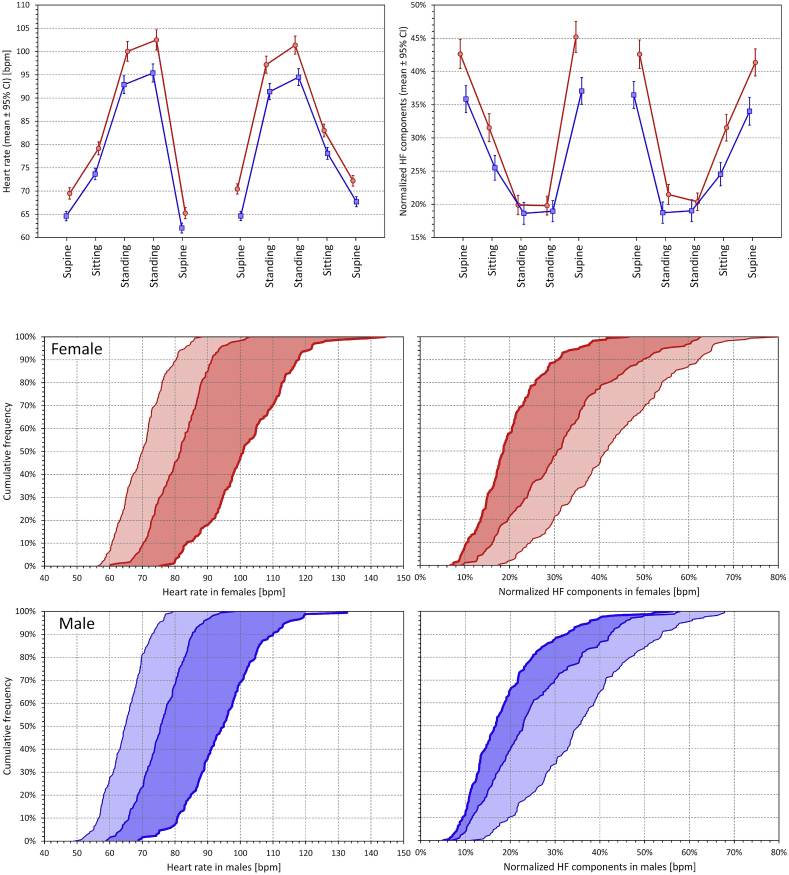
The top part shows measured values of heart rate (left panel) and of quasi-normalised high frequency HRV components (right panel) during pre-specified 5-min windows of the postural tests. In each panel, the left and right parts correspond to the postural tests 1 and 2, respectively. The positions are listed under the panels (the order of the two standing positions corresponds to their sequence within the tests – see the text for details). In each panel, the red and blue lines correspond to female and male subjects, respectively. The coloured dots show the means of the sex-specific sub-population, the error bars show the 95% confidence intervals of the means. The bottom part shows the cumulative frequency distributions of 5-min measurements of heart rate (left panels) and of quasi-normalised high frequency components (right panels) during initial supine positions (fine lines), sitting positions (middle-width lines) and final 5-min of standing positions (bold lines). The data presented are the intra-subject averages of both postural tests. The read and blue panels correspond to female and male sub-populations, respectively. Light and dark shaded areas highlight the shifts between supine and sitting positions, and between the sitting and standing positions, respectively. Note that the extent of dark shaded areas is similar between the sex groups for heart rate but not for quasi-normalised high-frequency HRV components.

The top part of Fig. 2 shows these data graphically for heart rate and nHF values. It demonstrates that while the heart rate reaction was similar in both sex groups (albeit starting from a different baseline level), the nHF reaction was very different in females and males. Consistent with previous reports [8,9] females showed larger nHF values at supine baseline. Nevertheless, the reduction of nHF by standing was more pronounced in females and during standing periods, the sex groups showed either little (during the middle 5-min period of standing at test 2) or no statistically significant difference in nHF (during all other standing 5-min periods).

The sex difference in nHF reaction is shown graphically by the cumulative distributions presented in the bottom part of Fig. 2. While the intra-individual supine → sitting → standing changes in heart rate show similar distribution shift in females and males, the corresponding distribution shifts in nHF are substantially different, especially concerning the sitting → standing changes.

**Fig. 3:**
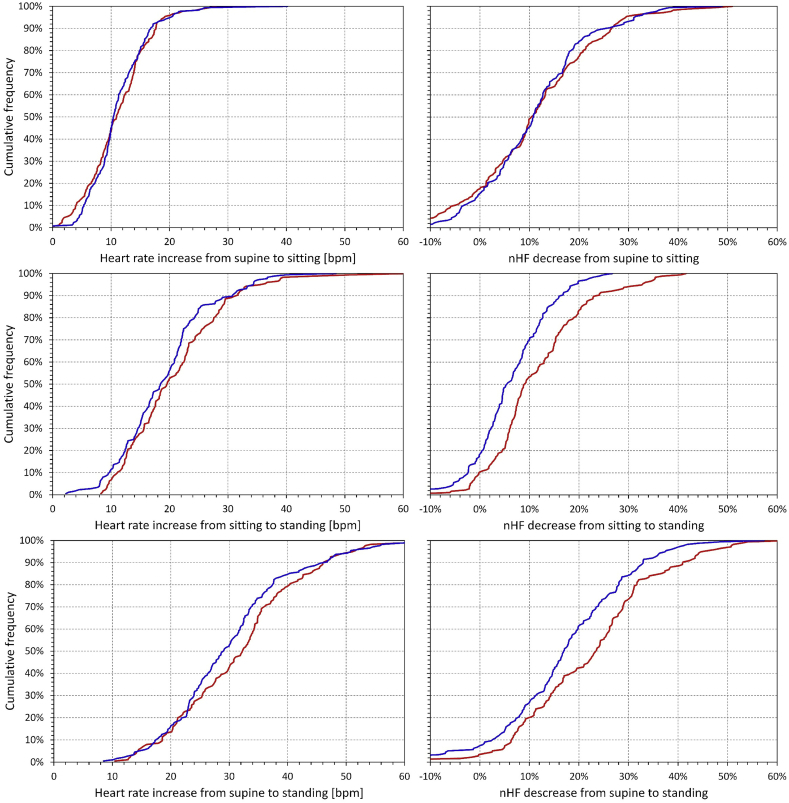
Cumulative distributions of changes of heart rate (left panels) and of quasi-normalised high frequency HRV components (right panels) during the positional changes from supine to sitting (top row panels), sitting to standing (middle row panels) and supine to standing (bottom row panels). The data presented are the changes of intra-subject averages of both postural tests; the data of the standing positions are taken from the final 5-min measurements. In each panel, the red and blue lines correspond to the female and male sub-populations, respectively.

This was further confirmed by the cumulative distribution of intra-individual heart rate and nHF changes from 5-min windows of initial supine positions to the sitting position and final 5-min windows of the standing position (intra-individual averages of both tests) are shown in Fig. 3. While there was no statistically significant difference between the distribution of the heart rate differences in females and males, the decrease in nHF was not statistically different between the sexes for the position change from supine to sitting but is was significantly larger in females compared to males for the change from sitting to standing (p < 0.001) and consequently also for the change from supine to standing (p < 0.001).

### 3.4 Covariates

**Fig. 4:**
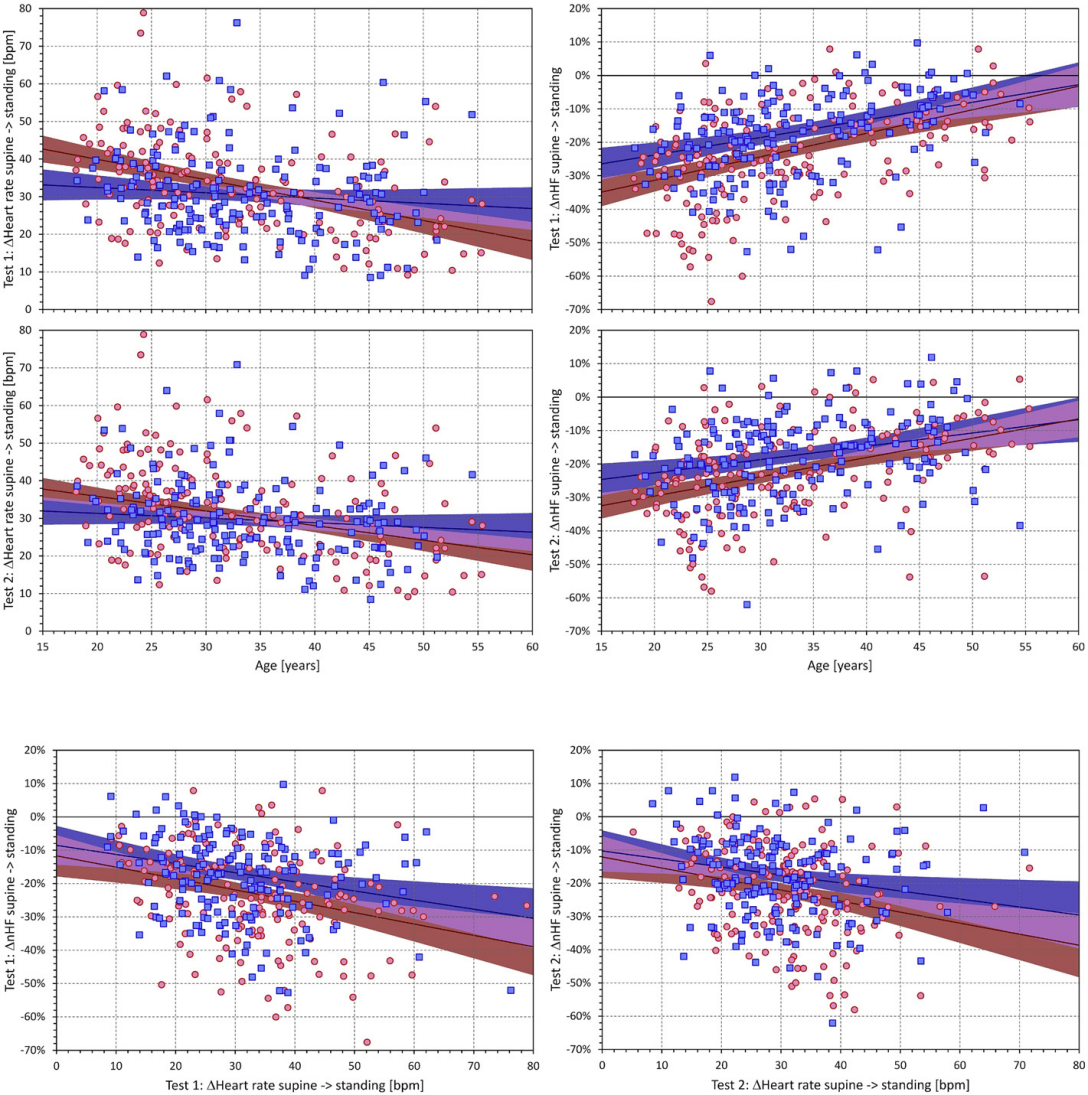
The top part shows scatter diagrams of age dependency of changes of heart rates (left panels) and of quasi-normalised high frequency HRV components (right panels) from supine to standing (final 5-min measurements) positions. The two rows of panels of the top part correspond to the postural tests 1 and 2, respectively. The bottom part shows the scatter diagrams of the dependency between heart rate changes and of quasi-normalised high frequency HRV component changes from supine to standing (final 5-min measurements) positions. The left and right panels of the bottom part correspond to the postural tests 1 and 2, respectively. In each panel, the red circles and blue squares correspond to female and male study subjects, respectively. The red and blue bold lines show the linear regressions of the displayed data in female and male sub-populations, respectively. The red and blue shaded areas show the 95% confidence bands of the linear regressions, the violet areas show the overlap of the confidence bands of both sex-specific regressions.

As expected, both intra-individual heart rate and nHF changes during postural changes showed relationship to age (top part of Fig. 4). Supine → standing heart rate change became lower with advancing age in both females (p < 0.001 in both tests) and in males (p = 0.026 and p = 0.021 in test 1, and test 2, respectively). Although the Figure (left panels of the top part) shows that the slopes in females were steeper than in males, the difference was not statistically significant.

The intra-individual nHF changes were also related to age (p < 0.001 in all comparisons). Contrary to the heart rate changes, the slopes in females were significantly steeper in females compared to males (p = 0.002 and p = 0.005 in tests 1 and 2, respectively).

As shown in the bottom part of Fig. 4, the intra-individual supine → standing changes in nHF were also related to the corresponding changes in heart rate (p < 0.001 for both comparisons in females, p = 0.005 and p = 0.03 for tests 1 and 2 in males, respectively). The corresponding slopes were also significantly different between females and males (p = 0.001 and p = 0.002 for tests 1 and 2, respectively). However, multivariable linear regression involving both age and the intra-individual heart rate changes as independent variables showed that age was the main determinant of nHF changes (p < 0.001 in all comparisons) with only modestly significant influence of heart rate changes in test 1 (p = 0.01 and p = 0.03 for females and males, respectively) and with no significant influence of heart rate changes in test 2.

## 4 Discussion

The study provides two potentially unexpected observations. Firstly, in healthy middle-aged subjects, postural change from supine to unsupported sitting leads to averaged heart rate increases of approximately 10 bpm whilst the change from supine to unsupported standing increases heart rate on average by approximately 30 bpm.

Secondly, while the extent of heart rate increases during postural change from supine to standing is not different between females and males, HRV spectral analysis shows surprisingly larger decrease of normalised HF components in females compared to males. At supine rest, females show larger proportion of nHF components but when challenged with unsupported standing over 10–15 min, they decrease their nHF components more than males so that the nHF difference between the sexes disappears.

Both these observations were also confirmed when studying the heart rate and HRV indices in the separate provocative tests of the individual days (i.e. without averaging the results of different provocative tests in each subject). The results of these separate analyses (not shown here) were practically identical in all the different baseline days of the source clinical study.

The shifts in the HRV components towards LF modulations on postural challenge have been known for decades [17]. It became customary to interpret these shifts in terms of cardiac autonomic status although the association between HRV indices and the autonomic tone is complex and not without limitations [18,19]. Undoubtedly, postural changes lead to changes in the autonomic status with shifts towards sympathetic dominance on standing. Hence, if interpreting the observed nHF changes using the customary model that links HF to primarily vagal modulations and LF to combined vagal and sympathetic modulations [16], the data might propose not only that the described postural change from supine to standing leads to more pronounced sympathetic increase in females compared to males but also that the increased autonomic challenge imposed by standing eliminates the sex difference.

In both human and other mammals, sympathetic system is frequently characterised as responsible for ‘fight or flight’ reactions while the vagal system has mainly calming role responsible for organism recuperation during rest [20]. The baseline nHF sex difference and the more pronounced change due to physical provocation in females might therefore be the autonomic conditioning basis for the sex differences in social behaviour and stress responses [21,22] in which the ‘fight or flight’ reactions are more pronounced in males while female reactions are more of the ‘tend-and-befriend’ category exhibited in response to threatening situations. Such sex-specific behaviour is relevant for the protection of the social group and for offspring preservation. The female ‘tend-and-befriend’ reactions have also been described as persistent maternal characteristics unrelated to mental stress [23]. This is also well in agreement with our observations since we found statistically higher nHF values in females not only in supine but also in sitting positions, i.e. after a change with only a mild sympathetic increase. More profound sympathetic stress of somewhat prolonged standing eliminated the sex difference. This might suggest that compared to males, female ‘fight or flight’ responses might require stronger initiation triggers which again appears to agree with population data on sex differences in physical conflicts [24,25]. It is thus plausible to speculate that the differences in autonomic regulation contribute to the known sex disparities in inter-personal interactions and social behaviour.

Since baseline sympathetic tone is also known to be potentially proarrhythmic, especially in patients without pre-existing repolarisation abnormalities [26], we might also speculate that the sex differences in the susceptibility to ventricular fibrillation [27,28] are contributed not only by sex hormones [27] but also by the differences in the baseline cardiac autonomic balance. This is also in agreement with our observation that the nHF sex differences were mainly present in younger age groups (note the convergence of the regression lines in the right panels of the top part of Fig. 4). Similarly, our observations of the sex differences in nHF reduction in response to substantial challenge might be relevant in interpreting the observations that among long QT syndrome type II patients, mainly females are at risk of arrhythmias induced by stimuli (e.g. abrupt awakening) that lead to substantial heart rate increases [29]. Nevertheless, it also needs to be added that since the majority of subjects of our study were relatively young and without any cardiac abnormality detectable at standard clinical screening [14], any speculations linking our observations to arrhythmic susceptibility need to be carefully interpreted with caution.

### 4.1 Limitations

Limitations of our study and data analysis also need to be considered. The data available for the investigation originated in a study of healthy subjects. We are therefore unable to comment on whether similar sex differences also exist in patients with cardiac and/or autonomic abnormalities (e.g. diabetic patients [30]). Likewise, since the data were obtained in primarily middle-aged adults, we are unable to comment on sex differences in healthy children or in the advanced age elderly [31,32]. The provocative tests were of physical nature. It would be interesting to investigate whether other challenges, e.g. mental stress, lead to similar sex differences. Postural provocation does not lead to very pronounced autonomic changes such as those known from exercise tests with high energy outputs. We do not have data to comment on such experiments. We also have no data on physical training of the investigated subjects. Nevertheless, since the study participants were recruited from a pool of volunteers available for clinical pharmacology investigations, it seems unlikely that physical training status was markedly different between the sex groups. Since no hormonal measurements were available from the source study, we are unable to comment on the possible influences of sex hormones to the observations made. Similarly, we are unable to comment on the influence of menstrual cycle in the investigated females, although some HRV differences in different phase of menstrual cycle have previously been reported by some authors [33] albeit disputed by others [34]. In the HRV investigation, we used standard spectral analysis and based the analyses on quasi-normalised frequency components since these are little influenced by the underlying heart rate (because of the heart rate dependency, we have not used the LF/HF ratio). Since the protocol of the source study permitted a window of up to 20 s to achieve the postural changes, we are unable to reproduce the interesting results of the speed of immediate heart rate recovery after standing that has previously been shown to predict all-cause mortality in general population of the elderly [35]. Finally, we cannot presently comment on the sex comparison of the short-term non-linear HRV indices [36]. We have not used these indices since their physiologic interpretation is less established compared to the spectral components.

## 5 Conclusion

Despite these limitations, the analysed data show that in both females and males, change of posture from resting supine to unsupported standing leads to surprising large elevation of heart rate with an average increase of around 30 bpm. Despite the similarity of the heart rate increase in both sexes, HRV analysis confirmed substantial differences in the high and low frequency heart rate modulations in females and males. In females, higher proportion of high frequency modulations was observed both during resting supine and sitting positions. Nevertheless, the change from sitting to standing position led to significantly larger suppression of relative high frequency modulation in females. At standing, the HRV spectral differences between sexes were abolished.

## Funding

Supported in part by the British Heart Foundation New Horizons Grant NH/16/2/32499 and by Ministry of Health, Czech Republic, conceptual development of research organization (Grant FNBr/65269705.)

## Declaration of competing interest

None declared.

## References

[1] Styles K., Sapp J., Gardner M., Gray C., Abdelwahab A., MacIntyre C., Gao D., Al-Harbi M., Doucette S., Theriault C., Parkash R. (2017). The influence of sex and age on ventricular arrhythmia in a population-based registry. Int. J. Cardiol..

[2] Chorin E., Hochstadt A., Viskin S., Rozovski U., Havakuk O., Baranchuk A., Enriquez A., Strasberg B., Guevara-Valdivia M.E., Márquez M.F., González-Pacheco H., Hasdemir C., Rosso R. (2017). Female gender as independent risk factor of torsades de pointes during acquired atrioventricular block. Heart Rhythm.

[3] Stramba-Badiale M., Spagnolo D., Bosi G., Schwartz P.J. (1995). Are gender differences in QTc present at birth? MISNES investigators. Multicenter Italian study on neonatal electrocardiography and sudden infant death syndrome. Am. J. Cardiol..

[4] Stramba-Badiale M., Locati E.H., Martinelli A., Courville J., Schwartz P.J. (1997). Gender and the relationship between ventricular repolarization and cardiac cycle length during 24-h Holter recordings. Eur. Heart J..

[5] Zareba W., Moss A.J., Locati E.H., Lehmann M.H., Peterson D.R., Hall W.J., Schwartz P.J., Vincent G.M., Priori S.G., Benhorin J., Towbin J.A., Robinson J.L., Andrews M.L., Napolitano C., Timothy K., Zhang L., Medina A. (2003). Modulating effects of age and gender on the clinical course of long QT syndrome by genotype. J. Am. Coll. Cardiol..

[6] Hohnloser S.H., Klingenheben T., van de Loo A., Hablawetz E., Just H., Schwartz P.J. (1994). Reflex versus tonic vagal activity as a prognostic parameter in patients with sustained ventricular tachycardia or ventricular fibrillation. Circulation.

[7] Porta A., Girardengo G., Bari V., George A.L., Brink P.A., Goosen A., Crotti L., Schwartz P.J. (2015). Autonomic control of heart rate and QT interval variability influences arrhythmic risk in long QT syndrome type 1. J. Am. Coll. Cardiol..

[8] Britton A., Shipley M., Malik M., Hnatkova K., Hemingway H., Marmot M. (2007). Changes in heart rate and heart rate variability over time in middle-aged men and women in the general population (from the Whitehall II Cohort Study). Am. J. Cardiol..

[9] Huikuri H.V., Pikkujämsä S.M., Airaksinen K.E., Ikäheimo M.J., Rantala A.O., Kauma H., Lilja M., Kesäniemi Y.A. (1996). Sex-related differences in autonomic modulation of heart rate in middle-aged subjects. Circulation.

[10] Kuch B., Hense H.W., Sinnreich R., Kark J.D., von Eckardstein A., Sapoznikov D., Bolte H.D. (2001). Determinants of short-period heart rate variability in the general population. Cardiology.

[11] Yamasaki Y., Kodama M., Matsuhisa M., Kishimoto M., Ozaki H., Tani A., Ueda N., Ishida Y., Kamada T. (1996). Diurnal heart rate variability in healthy subjects: effects of aging and sex difference. Am. J. Physiol..

[12] Evans J.M., Ziegler M.G., Patwardhan A.R., Ott J.B., Kim C.S., Leonelli F.M., Knapp C.F. (2001). Gender differences in autonomic cardiovascular regulation: spectral, hormonal, and hemodynamic indexes. J. Appl. Physiol..

[13] Schwartz P.J., Zaza A., Locati E., Moss A.J. (1991). Stress and sudden death. The case of the long QT syndrome. Circulation.

[14] ICH Guideline (2001). Safety pharmacology studies for human pharmaceuticals S7A. Fed. Regist..

[15] Malik M., Andreas J.O., Hnatkova K., Hoeckendorff J., Cawello W., Middle M., Horstmann R., Braun M. (2008). Thorough QT/QTc study in patients with advanced Parkinson’s disease: cardiac safety of rotigotine. Clin. Pharmacol. Ther..

[16] Task Force of the European Society of Cardiology and the North American Society of Pacing and Electrophysiology (1996). Heart rate variability - standards of measurement, physiological interpretation, and clinical use. Circulation.

[17] Pomeranz B., Macaulay R.J.B., Caudill M.A., Kutz I., Adam D., Gordon D., Kilborn K.M., Barger A.C., Shannon D.C., Cohen R.J., Benson H. (1985). Assessment of autonomic function in humans by heart rate spectral analysis. Am. J. Physiol..

[18] Malik M., Hnatkova K., Huikuri H., Lombardi F., Schmidt G., Zabel M. (2019). CrossTalk proposal: heart rate variability is a valid measure of cardiac autonomic responsiveness. J. Physiol..

[19] Malik M., Hnatkova K., Huikuri H., Lombardi F., Schmidt G., Zabel M. (2019). CrossTalk rebuttal. J. Physiol..

[20] Jansen A.S., Nguyen X.V., Karpitskiy V., Mettenleiter T.C., Loewy A.D. (1995). Central command neurons of the sympathetic nervous system: basis of the fight-or-flight response. Science.

[21] Taylor S.E., Klein L.C., Lewis B.P., Gruenewald T.L., Gurung R.A., Updegraff J.A. (2000). Biobehavioral responses to stress in females: tend-and-befriend, not fight-or-flight. Psychol. Rev..

[22] von Dawans B., Ditzen B., Trueg A., Fischbacher U., Heinrichs M. (2019). Effects of acute stress on social behavior in women. Psychoneuroendocrinology.

[23] Tifferet S., Manor O., Constantini S., Friedman O., Elizur Y. (2011). Sex differences in parental reaction to pediatric illness. J. Child Health Care.

[24] Lowry R., Powell K.E., Kann L., Collins J.L., Kolbe L.J. (1998). Weapon-carrying, physical fighting, and fight-related injury among U.S. adolescents. Am. J. Prev. Med..

[25] Schnitzer S., Bellis M.A., Anderson Z., Hughes K., Calafat A., Juan M., Kokkevi A. (2010). Nightlife violence: a gender-specific view on risk factors for violence in nightlife settings: a cross-sectional study in nine European countries. J. Interpers Violence.

[26] Stramba-Badiale M., Lazzarotti M., Facchini M., Schwartz P.J. (1994). Malignant arrhythmias and acute myocardial ischemia: interaction between flecainide and the autonomic nervous system. Am. Heart J..

[27] Linde C., Bongiorni M.G., Birgersdotter-Green U., Curtis A.B., Deisenhofer I., Furokawa T., Gillis A.M., Haugaa K.H., Lip G.Y.H., Van Gelder I., Malik M., Poole J., Potpara T., Savelieva I., Sarkozy A. (2018). Sex differences in cardiac arrhythmia: a consensus document of the european heart rhythm association, endorsed by the heart rhythm society and asia pacific heart rhythm society. Europace.

[28] Wellens H.J., Schwartz P.J., Lindemans F.W., Buxton A.E., Goldberger J.J., Hohnloser S.H., Huikuri H.V., Kääb S., La Rovere M.T., Malik M., Myerburg R.J., Simoons M.L., Swedberg K., Tijssen J., Voors A.A., Wilde A.A. (2014). Risk stratification for sudden cardiac death: current status and challenges for the future. Eur. Heart J..

[29] Schwartz P.J., Priori S.G., Spazzolini C., Moss A.J., Vincent G.M., Napolitano C., Denjoy I., Guicheney P., Breithardt G., Keating M.T., Towbin J.A., Beggs A.H., Brink P., Wilde A.A., Toivonen L., Zareba W., Robinson J.L., Timothy K.W., Corfield V., Wattanasirichaigoon D., Corbett C., Haverkamp W., Schulze-Bahr E., Lehmann M.H., Schwartz K., Coumel P., Bloise R. (2001). Genotype-phenotype correlation in the long-QT syndrome: gene-specific triggers for life-threatening arrhythmias. Circulation.

[30] Hansen C.S., Færch K., Jørgensen M.E., Malik M., Witte D.R., Brunner E.J., Tabák A.G., Kivimäki M., Vistisen D. (2019). Heart rate, autonomic function, and future changes in glucose metabolism in individuals without diabetes: the Whitehall II cohort study. Diabetes Care.

[31] Andršová I., Hnatkova K., Helánová K., Šišáková M., Novotný T., Kala P., Malik M. (2019). Individually rate corrected QTc intervals in children and adolescents. Front. Physiol..

[32] Reardon M., Malik M. (1996). Changes in heart rate variability with age. Pacing Clin. Electrophysiol..

[33] Sato N., Miyake S., Akatsu J., Kumashiro M. (1995). Power spectral analysis of heart rate variability in healthy young women during the normal menstrual cycle. Psychosom. Med..

[34] Leicht A.S., Hirning D.A., Allen G.D. (2003). Heart rate variability and endogenous sex hormones during the menstrual cycle in young women. Exp. Physiol..

[35] McCrory C., Berkman L.F., Nolan H., O’Leary N., Foley M., Kenny R.A. (2016). Speed of heart rate recovery in response to orthostatic challenge. Circ. Res..

[36] Sassi R., Cerutti S., Lombardi F., Malik M., Huikuri H.V., Peng C.K., Schmidt G., Yamamoto Y. (2015). Advances in heart rate variability signal analysis: joint position statement by the e-Cardiology ESC Working Group and the European Heart Rhythm Association co-endorsed by the Asia Pacific Heart Rhythm Society. Europace.

