# Supplementary material for: Sex differences in heart rate responses to postural provocations
Source: Int J Cardiol. 2019 Dec 15;297:126–34. doi: 10.1016/j.ijcard.2019.09.044 (PMC6926477; doi:10.1016/j.ijcard.2019.09.044)
Supplement: Multimedia component 1 [file mmc1.docx]

Table 1

Heart rate and heart rate variability measurements

|  |  | **Heart rate** | **Total Power** | **LF Power** | **HF power** | **nHF** |
| --- | --- | --- | --- | --- | --- | --- |
| **Test 1** | | | | | | |
| Supine | F | 69.5±8.2 | 2532±2154 | 783±678 | 759±1206 | 0.426±0.146 |
|  | M | 64.6±6.8 | 2406±1604 | 824±709 | 521±541 | 0.359±0.138 |
|  | p | <0.001 | NS | NS | NS | <0.001 |
| Sitting | F | 79.2±9.3 | 2215±1496 | 777±590 | 409±506 | 0.315±0.140 |
|  | M | 73.7±8.4 | 2457±1505 | 963±714 | 341±326 | 0.255±0.125 |
|  | p | <0.001 | 0.034 | 0.001 | NS | <0.001 |
| Standing | F | 100.0±14.1 | 1082±861 | 434±496 | 100±102 | 0.199±0.097 |
|  | M | 92.9±12.8 | 1445±987 | 605±524 | 137±141 | 0.186±0.111 |
|  | p | <0.001 | <0.001 | <0.001 | 0.009 | NS |
| Standing | F | 102.5±14.5 | 1119±832 | 434±435 | 105±100 | 0.198±0.095 |
|  | M | 95.4±13.1 | 1453±1106 | 622±546 | 150±179 | 0.190±0.107 |
|  | p | <0.001 | 0.002 | <0.001 | 0.012 | NS |
| Supine | F | 65.3±8.0 | 3264±3268 | 1031±1058 | 1160±1892 | 0.452±0.155 |
|  | M | 62.0±7.1 | 2683±2075 | 953±860 | 649±880 | 0.371±0.135 |
|  | p | <0.001 | NS | NS | 0.012 | <0.001 |
| **Test 2** | | | | | | |
| Supine | F | 70.4±7.4 | 2361±1961 | 669±528 | 659±1017 | 0.426±0.144 |
|  | M | 64.6±6.7 | 2241±1424 | 743±574 | 501±559 | 0.365±0.135 |
|  | p | <0.001 | NS | 0.033 | NS | <0.001 |
| Standing | F | 97.2±12.3 | 1085±658 | 401±335 | 112±145 | 0.215±0.101 |
|  | M | 91.4±11.7 | 1345±768 | 555±424 | 134±181 | 0.187±0.107 |
|  | p | <0.001 | <0.001 | <0.001 | 0.206 | 0.014 |
| Standing | F | 101.4±13 | 1007±697 | 376±345 | 96±94 | 0.204±0.088 |
|  | M | 94.5±12.2 | 1303±884 | 533±440 | 133±213 | 0.190±0.111 |
|  | p | <0.001 | 0.001 | <0.001 | 0.039 | NS |
| Sitting | F | 83.0±9.2 | 1618±1186 | 563±471 | 298±340 | 0.315±0.134 |
|  | M | 78.1±8.7 | 1762±1037 | 672±450 | 246±259 | 0.245±0.118 |
|  | p | <0.001 | 0.024 | 0.001 | NS | <0.001 |
| Supine | F | 72.2±7.7 | 2013±1717 | 648±574 | 550±662 | 0.414±0.136 |
|  | M | 67.7±7.3 | 2001±1563 | 699±621 | 428±588 | 0.340±0.141 |
|  | p | <0.001 | NS | NS | NS | <0.001 |

For different 5-minute windows of both postural provocative tests, the table shows heart rate and spectral heart rate variability measurements in female (F) and male (M) study sub-populations. The data are mean ± standard deviation and for each 5-minute windows, p-value (p) of the comparison between female and male subjects is shown. The 5-minute windows of the standing position are shown in sequence as they occurred during the tests (see the text for details).
